# Co-creation process of an intervention to implement a multiparameter point-of-care testing device in a primary healthcare setting for non-communicable diseases in Peru

**DOI:** 10.1186/s12913-024-10809-3

**Published:** 2024-03-29

**Authors:** Leonardo Albitres-Flores, Silvana Perez-Leon, Antonio Bernabe-Ortiz, Janeth Tenorio-Mucha, Maria Kathia Cardenas, Beatrice Vetter, Elvis Safary, Ricardo Gamboa, Vicente Cordova, Reena Gupta, Andrew Moran, David Beran, María Lazo-Porras

**Affiliations:** 1 CRONICAS Centre of Excellence in Chronic Diseases, Universidad Peruana Cayetano Heredia, Lima, Peru; 2 FIND, Geneva, Switzerland; 3 Global Health Center, Universidad Peruana Cayetano Heredia, Tumbes, Peru; 4 Dirección Regional de Salud de Tumbes, Tumbes, Peru; 5 Resolve to Save Lives, New York, NY USA; 6 Division of General Internal Medicine, San Francisco General Hospital, University of California, San Francisco, USA; 7 Division of Tropical and Humanitarian Medicine, Geneva University Hospitals & University of Geneva, Geneva, Switzerland

**Keywords:** Point-of-care testing, Community-based Participatory Research, Primary Health Care, Noncommunicable diseases

## Abstract

**Background:**

Point-of-care testing (POCT) devices are diagnostic tools that can provide quick and accurate results within minutes, making them suitable for diagnosing non-communicable diseases (NCDs). However, these devices are not widely implemented in healthcare systems and for this reason is relevant to understand the implementation process.

**Aim:**

To describe the process and define a strategy to implement a multiparameter POCT device for diagnosing and managing NCDs in one region of Peru.

**Methods:**

A descriptive and non-experimental study, using the participatory methodologies of co-creation process. It was conducted in one region of Peru (Tumbes) to design an intervention for implementing a multiparameter POCT device. Two co-creation sessions were conducted involving five groups: community members, primary healthcare workers, these groups in both rural and urban settings, and regional decision-makers. These sessions included activities to understand patient journeys in receiving care for NCDs, identify facilitators and barriers to POCT devices usage, and define an implementation strategy for POCT devices in both rural and urban settings of Tumbes. The research team analysed the data and summarized key topics for discussion after each session.

**Results:**

A total of 78 participants were enrolled across the five groups. Among community members: 22.2% had only diabetes, 24.1% had only hypertension, and 18.5% had both diagnoses. In the patient journey, community members mentioned that it took at least three days to receive a diagnosis and treatment for an NCD. Most of the participants agreed that the POCT devices would be beneficial for their communities, but they also identified some concerns. The strategy for POCT devices implementation included healthcare workers training, POCT devices must be placed in the laboratory area and must be able to perform tests for glucose, glycated haemoglobin, cholesterol, and creatinine. Advertising about POCT devices should be displayed at the healthcare centres and the municipality using billboards and flyers.

**Conclusions:**

The co-creation process was useful to develop strategies for the implementation of multiparameter POCT devices for NCDs, involving the participation of different groups of stakeholders guided by moderators in both, rural and urban, settings in Peru.

**Supplementary Information:**

The online version contains supplementary material available at 10.1186/s12913-024-10809-3.

## Introduction

Worldwide, non-communicable diseases (NCDs) are a major public health concern [1]. Principal NCDs, including cardiovascular diseases, diabetes, chronic renal disease and cancer, account for approximately 74% of all global deaths, causing 41 million deaths annually [2, 3]. Early detection and appropriate monitoring and management of NCDs are essential actions to reduce morbidity, mortality, and the burden of NCDs, especially in low- and middle-income countries (LMICs) [4].

People living with NCDs often face barriers to receive an appropriate diagnosis, as well as the necessary resources to manage their condition effectively. In LMICs, disparities in social, geographic, and economic factors exacerbate the lack of access to healthcare [5]. Additionally, many people living with NCDs cannot afford the cost of healthcare [6]. At the primary healthcare level, there is often insufficient trained health personnel and limited available services [7]. Furthermore, there are limitations in the laboratory services, including scarce testing equipment and insufficient supplies for the most basic clinical chemistry parameters required to manage cardiometabolic conditions as recommended by the World Health Organization [3]. These limitations result in patients being referred to higher levels of care or private laboratories for access to adequate diagnosis and management. Such barriers can lead to increased morbidity and diminished quality of life [8].

Point-of-care testing (POCT) devices are diagnostic tools available for clinical chemistry testing that are easy to use and can provide quick and accurate results within minutes at the location where the patient is being treated [9, 10]. The POCT devices can be used for both the diagnosis of NCDs and the monitoring of their management at the point-of-care. Multiparameter POCT devices allows for the process of different tests in a single machine, which can offer a more comprehensive view of the patient’s condition, saving time and resources by reducing the need for multiple separate laboratory tests or devices [10]. Moreover, these tests are typically performed using fingerstick capillary blood, eliminating the requirement for venous blood draw and serum/plasma preparation [11]. This can improve the patient’s involvement in his/her treatment and alleviate barriers associated with conducting all clinically recommended tests [12].

The POCT devices can be used in different settings, including rural or remote communities. Although currently available POCT devices may still have technical limitations concerning infrastructure requirements, training needs and test procedures, they still often a valuable opportunity to bring testing closer to the patient, particularly for many primary healthcare centres (PHCs) [13–15]. The implementation of POCT devices is a strategy with the potential to reduce the burden of NCDs and improve access to diagnosis at the primary healthcare level, as well as monitoring of NCDs [16].

The implementation of POCT devices in a PHC setting may encounter various barriers [16], and a co-creation process can be used to determine the implementation strategies for interventions in different PHC settings [17]. A co-creation involves the development of interventions with the collaborative participation of all parties involved in the process [18]. This approach can take different forms, such as conducting workshops or interviews with patients and healthcare workers to gather information about their specific needs related to NCDs. The objective of this research is to describe the process and to define a strategy for implementing a multiparameter POCT device for the diagnosis and management of NCDs in a region of Peru. However, an effective implementation strategy needs to be adapted to the context and considers the voices of different stakeholders to ensure future sustainability and scalability [17]. Consequently, this study presents the description and findings of a co-creation process developed with the aim of implementing multiparameter POCT devices in both rural and urban settings in a region of Peru.

## Methods

### Study design

A descriptive and non-experimental study, using the participatory methodologies of co-creation process. It was conducted to design an intervention to implement a multiparameter POCT device. Two sessions of co-creation workshops were conducted with each group of stakeholders, including community members, primary healthcare workers (PHCW) and regional decision-makers.

### Settings

This study took place in two sites, one rural and one urban, in Tumbes, a coastal region located in the northern part of Peru. The rural site is located 18 km away from the Regional Hospital of Tumbes, while the urban site is 7 km away. Tumbes has a high prevalence of cardiovascular diseases compared to the national average. According to the National Demographic and Health Survey (ENDES), in 2020 the prevalence of hypertension was 22.1% and obesity was 40.1% in the region. Additionally, Tumbes had a prevalence of 10.3% for diabetes mellitus and 12.9% for chronic kidney disease [19]. Undiagnosed diabetes prevalence was 41.3% [20]. Furthermore, there were no differences in the incidence of diabetes between the urban and rural groups [19].

### Participants and recruitment

The participants in the co-creation process included community members, PHCW, and decision-makers. Community members were individuals aged 18 years or above who have lived in the area for at least one year and with or without diagnosis of NCDs. The PHCW included physicians, nurses, obstetricians, technicians or workers who provided healthcare services for the community in the selected sites. Regional decision-makers were representatives of the Regional Health Directorate of Tumbes, and they were invited considering whether their functions were related to the care of patients with non-communicable diseases. A local study coordinator facilitated meetings with decision-makers at the Regional Health Directorate and with the PHCW at each site. During the first meeting, one investigator explained the study objectives and defined the dates for the first co-creation session (October 18th -20th, 2022). The PHCW assisted in recruiting community members prioritizing those with NCDs, and also invited some community health workers. Community healthcare workers are volunteers who promote healthy lifestyles, preventive actions and health promotion within families in their communities [21]. They all provided a written informed consent before participating in the study.

### Sampling

This study did not have a predetermined sample size [18], but aimed to have approximately 30 participants in each workshop with community members, and the maximum number of participants in workshops with PHCW at each site. We invited six regional health decision makers (Regional Director of Health, network Executive Director, Regional Director of Epidemiology, Regional Director of Laboratory, heads of strategies: NCDs, Health of people, Health Promotion, Comprehensive Health Care) to co-creation activities, some of them could have more than one position. We applied purposive sampling, which has been used in previous experiences [17], to ensure better management of small groups.

### Procedures for planning co-creation

Based upon a scoping review assessing barriers and facilitators for implementing POCT devices for NCDs conducted by our research group [16]. We obtained this information to share and conduct the co-creation process with various groups of stakeholders.

The co-creation process engaged five groups of stakeholders. These included community members and PHCW, in both rural and urban settings. Additionally, one group of regional decision-makers because their decisions cover both rural and urban areas. A co-creation session was developed for each group of stakeholders in each workshop.

The co-creation sessions with community members had four moderators. These moderators had the following characteristics: a sociologist with experience in participatory methods and qualitative methodology and chronic diseases; an obstetrician who was familiar with the PHCW and the specific area; and two physicians, one with experience in co-creation field work, while both had knowledge in epidemiology. Coordination meetings among the moderators were held to discuss session activities, prepare the materials, and carry out a pilot. In the pilot the moderators met and simulated a co-creation workshop to measure the times necessary for each proposed activity, reorder and modify them.

### Development of co-creation activities

Different activities were planned to promote the participation of different stakeholders. The study objectives and procedures were explained to the participants, who were then asked to sign a voluntary informed consent form to participate in the co-creation sessions and consent to audio recording. Sociodemographic data was collected such as age, sex, and self-reported NCDs (arterial hypertension, diabetes, chronic kidney disease, coronary disease, among others). Subsequently, the participants were divided into small groups of approximately 8 people during the workshop (with a moderator per group), assigned in order of arrival. In the first session, each group was asked to design a “journey map” depicting the routine of patients in the healthcare centre, from their arrival to receiving a diagnosis and treatment for an NCD. This activity aimed to understand the process and time incurred by people with NCDs. Next, the moderators explained the concept of POCT device is and presented a summary of the results of the scoping review to raise awareness among the participants about POCT devices. Finally, a brainstorming activity was conducted, where the participants were asked about their opinion on the expected facilitators/advantages and barriers/disadvantages of the POCT devices. The moderators also explored strategies to improve the implementation of POCT devices for NCDs in their settings.

Between the sessions, the research team reviewed and discussed the results obtained from the first co-creation workshops and summarized topics to be discussed in the second co-creation session (see Data Analysis section). The criteria to select the topics were feasibility, the needs of the population, and the potential impact of the intervention components. The dates for the second co-creation session were coordinated with the health personnel in each site to avoid any scheduling conflicts with other local activities.

**Fig. 1:**
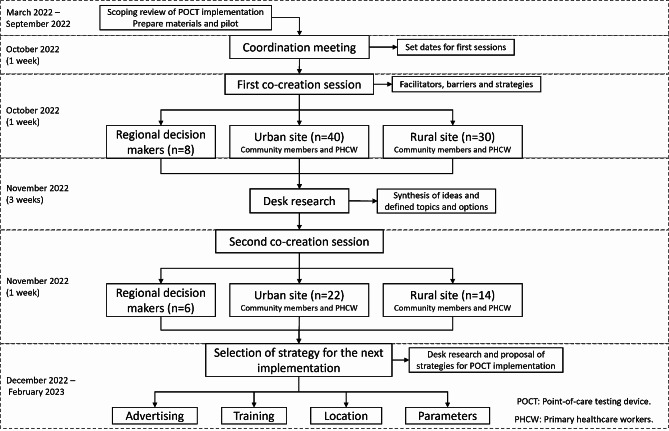
Flowchart of the co-creation process of strategies for point-of-care testing devices implementation

In the second co-creation session, the same participants were invited. The topics and strategies proposed in the first session were discussed with the participants in small groups of approximately 8 people, assigned in order of arrival. The topics discussed included advertising modalities and settings, training of healthcare personnel to use the POCT device, desired laboratory parameters for the device, and the location of the POCT devices (such as laboratory, doctor’s office, triage, and extramural). Participants were asked to prioritize the options for each topic and explain the reasons behind their decisions. Furthermore, during the activity about training, the moderators explored participants’ preferences regarding the modality (virtual vs. face-to-face) for training, types of PHCW that should be trained, duration of training, evaluation methods, and identifying best trainers. Activities related to training and desirable laboratory parameters were only carried out with PHCW and regional decision-makers because they were aware of the technical aspects of the processes **(**Fig. 1**)**.

### Data analysis

In the first co-creation workshop, the data collected from each group during the co-creation activities were synthesised separately by the research team and consolidated in several meetings. The journey maps created by each group were used to develop a general flowchart of the patient’s journey and estimate the time needed to navigate through the PHC system to receive care. Then, the most frequent ideas that emerged during the discussions on facilitators/advantages and barriers/disadvantages were identified by the research team using thematic analysis. No transcription or analysis with codes was carried out. They also discussed the feasibility and contributions of these ideas to the objective of the study. Strategies that involved other types of interventions or addressed issues unrelated to the objective of the study were discarded.

After the synthesizing the findings, the research team reviewed them to identify the main topics to be discussed during the second co-creation workshop, which included advertising modality and settings, health personnel training, desired parameters for the device, and the location of the POCT device. For each topic, the most important and feasible options that emerged during the first workshop were selected to be presented to the participants in the second co-creation session. After the second co-creation session, a hierarchical order of the selected choices from each small group was identified, taking into consideration the reasons behind their selection. These results were further discussed by the research team, and the most feasible and appropriate alternatives for implementation were identified.

After both co-creation sessions, the research team defined the intervention components considering the information provided by different stakeholders. Descriptive quantitative analysis was performed using STATA 17.0. No software was used for qualitative data analysis.

### Ethics

This study protocol was reviewed and approved by the Institutional Research Ethics Committee of the Universidad Peruana Cayetano Heredia (IRB00001014, dated September 8, 2022). Prior to their participation in the study, all participants provided informed consent by signing a consent form.

## Results

A total of 78 participants were enrolled in the study: 8 (10.3%) regional decision maker, 30 (38.5%) from the rural site and 40 (51.3%) from the urban site; including 7 and 9 PHCW in each site, respectively. In the urban and rural site, community health workers (*n* = 8) participated and actively collaborated in the co-creation workshops, all of them were women. Across all participants, the mean age was 57.1 years (SD: 15.4), and 63 (80.8%) were women. Among the community members (*n* = 54); 12 (22.2%) had only diabetes, 13 (24.1%) had only hypertension, and 10 (18.5%) had both diagnoses. Other characteristics of the participants can be reviewed in Supplementary Table 1.

### First co-creation workshop

**Fig. 2:**
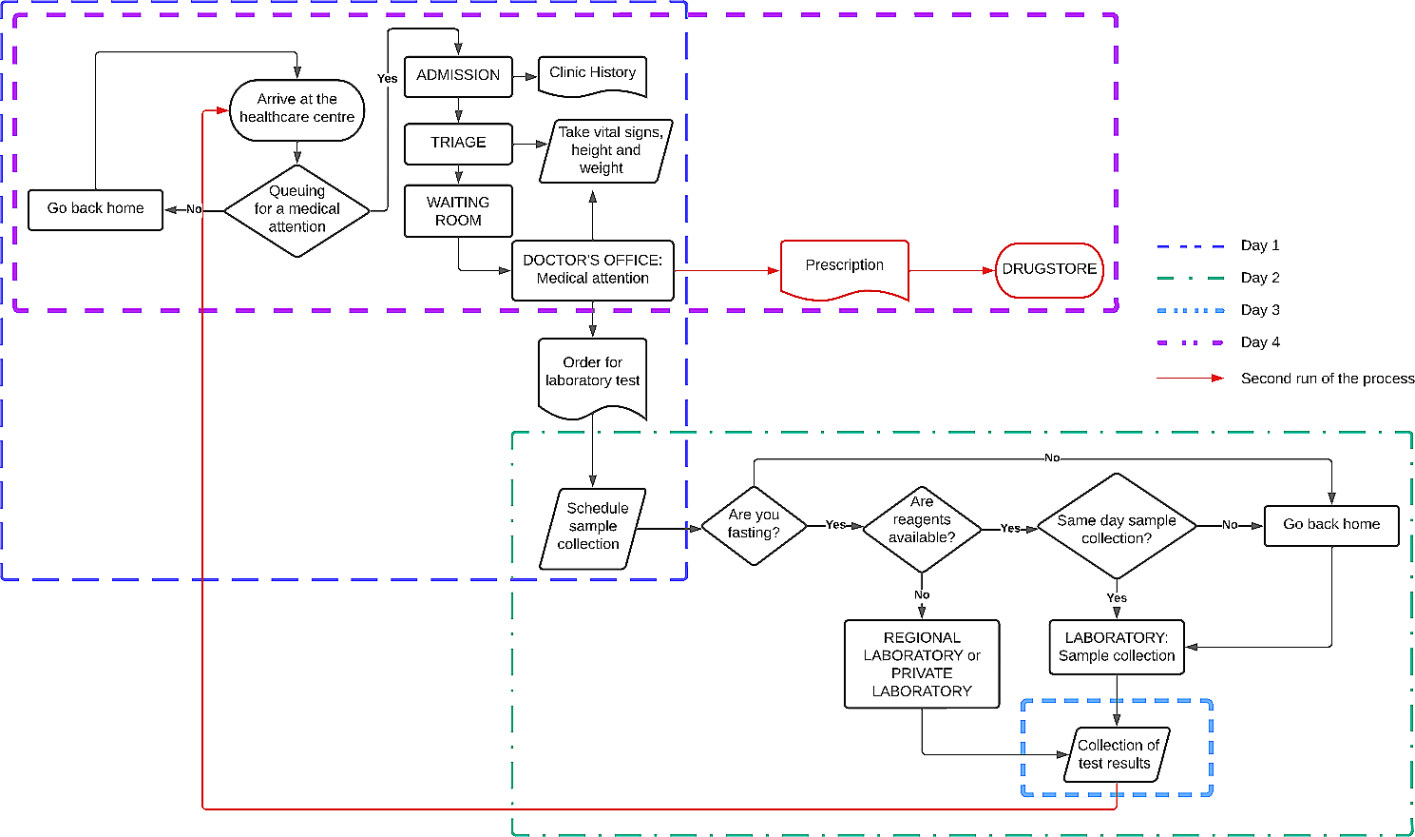
Flowchart of patient’s healthcare attention previous to the point-of-care testing device implementation

The first session workshops took place from 18th to 20th October 2022. During these workshops, participants developed a “journey map” of the patient’s routine at the healthcare centre. All groups presented a routine that required at least three days to receive treatment for their NCD (Fig. 2). Participants expressed concerns about the current care pathway, including a limited number of medical consultations per day, wait time for care, lack of laboratory test and shortage of medicines. Participants mentioned that they would prefer a one-day visit to the healthcare facility. The PHCW agreed with the idea of a one-day visit for patients including the laboratory tests. Physicians said, “It is possible that the first days will be more difficult, but then it will improve”. Community members and even healthcare personnel, were not familiar with the concept of POCT device, and for this reason, we had to explain how they work during the workshop activities. Older adults did not express themselves easily. Although there were few men, they always gave their opinion. The barriers mentioned by participants regarding the implementation of POCT devices included concerns about the safety of the device such as “it can be broken” and “it would take long time to fix it”. Other concerns were the accuracy of results compared to the current available laboratory tests, the need for training of healthcare personnel, the optimal location of the POCT devices within the facility in order to ensure their best use, advertising to the community to raise awareness about POCT devices, and the acceptability of the devices among the population (Table 1). All activities in the workshop lasted about 2 h.

**Table 1:** Perception of possible advantages, disadvantages, and strategies for the implementation of the POCT devices for NCDs

Advantages/Facilitators	Disadvantages/Barriers	Strategies
• POCT devices can be used in PHC level and health campaigns. Better for remote places. • They would not need to go to private laboratories. • Reduce waiting time. Results obtained quickly. • POCT devices can be user friendly. • POCT devices would reduce the out-of-pocket cost of patients.	• They may have a greater margin of error. • Lack of cost-benefit analysis. • Environmental conditions may not be suitable for POCT devices. • Limited number of samples for analysis at the same time. • Possible inaccessible consumables. • Requires training to use. • Security is required to prevent theft or damage. • Possible distrust in a very small device and would not like to have to repeat tests. • Need for constant maintenance and calibration of equipment	• Training and induction of health personnel. • Secure the device to prevent falls and/or theft. • Compare with conventional laboratory instruments.

POCT: Point-of-care testing; NCDs: Non-communicable diseases; PHC: Primary healthcare

The most frequently mentioned location for the POCT devices was the laboratory in the PHC facility, followed by the triage area and doctor’s office. The most frequently mentioned desired parameters were glucose, glycated haemoglobin, and lipid profile. The proposed modality of advertising to the community were loudspeakers, flyers, billboards, among others. Places that were more frequently proposed for advertising included the healthcare centre, the municipality, and the town square. Other topics mentioned in the workshop included questions about the number of samples from different patients tested simultaneously, as well as the need for POCT devices to have an internal battery due to constant power outages.

### Second co-creation workshop

The second workshop took place approximately one month after the first workshop (from 22nd to 24th November, 2022). The same participants were invited to attend, although nearly one-third of the participants from the community did not attend the second workshop. The most important topics identified in the previous workshop were presented to the participants. Regarding training for POCT device usage, both PHCW and regional decision-makers preferred to have theoretical-practical lessons, and they suggested to have the greatest number of PHCW to be trained. The training should be conducted in a single day. A user manual or video tutorials should be provided and a follow-up every 6 months should be carried out. Participants would like to obtain a certificate upon passing the training.

The most prioritized location was the laboratory because of the experience and expertise of personnel working there in taking and analysing blood samples. However, the laboratory is not always available due to the PHCW schedule or the number of patients that need testing. The second option for the location was the triage area due to the idea that patients can arrive at the doctor’s office with their test results, and they can save time, but triage do not have personnel trained in taking blood samples. Additionally, emergency area emerged as a new location in the urban groups because it is a service always available (see Table 2). When the possibility of community health workers using the POCT devices was discussed, most of the regional decision-makers mentioned that it is not possibly due to current regulations.

**Table 2:** Advantages and disadvantages for each possible point-of-care testing devices device location

Location	Advantages	Disadvantages
**Laboratory**	Personnel trained in sampling, more experienced in the use of similar equipment. Faster patient flow. Supplies available in the laboratory. Safe place for equipment. Access to the laboratory is always available by the staff.	There is already a queue to attend. Limited space. There are shifts without assigned personnel.
**Doctor office**	Immediate diagnostic opportunity. Trust in the doctor. The patient would be attended in a single consultation even if it takes longer. It would improve the flow with someone in the office to take the samples.	More time spent by the doctor. Possible refusal of the doctor to perform the procedure. It would delay the care of other patients. Doctors are not trained in taking blood samples. Some doctors are not friendly with their patients
**Triage**	It would help attract more patients. Patients would enter a medical consultation with their current results.	Equipment insecurity: risk of falls, theft. Staff time is limited. Personnel not trained in taking blood samples. Staff usually perform other functions in addition to triage.
**Extramural**	It would give greater coverage of the most remote population. Reach places without a fixed laboratory. Useful in medical campaigns, screening campaigns and other events.	Equipment insecurity: risk of falls, theft. Distrust of the accuracy of the results. May be affected by environmental conditions. There are no continuous activities, only sporadic medical campaigns.
**Emergency**	Quick attention and immediate results. Useful for management of severe patients.	*No disadvantages mentioned.

The main modality proposed in both rural and urban settings for raising awareness was the use of loudspeakers to reach remote settings, flyers and placement of billboards on commonly visited places such as the healthcare centre or the municipality (Table 3). The prioritised parameters were glucose, glycated haemoglobin, cholesterol, triglycerides, urea/creatinine, due to their usefulness in controlling and managing NCDs (Table 4). In the rural facility, only glucose and lipid profile tests were usually available, while in the urban facility, in addition to the previous tests, creatinine test was also available. In both facilities, glycated haemoglobin was not available.

**Table 3:** Modality and settings for awareness raising during the point-of-care testing device implementation

Regional decision-makers	Urban setting		Rural setting	
	Patients	Primary healthcare workers	Patients	Primary healthcare workers
**Modality**				
1. Social networks	5. Door to door	4. Billboards	3. Megaphone advertising	1. Social networks
2. Local TV and radio	6. Flyer	6. Flyer	1. Social networks	3 Megaphone advertising
3. Megaphone advertising	3. Megaphone advertising	3. Megaphone advertising	5. Door to door	4. Billboards
4. Billboards	4. Billboards		6. Calls	6. Flyer
**Places**				
1. Markets	1. Markets	2. Healthcare centre	2. Healthcare centre	2. Healthcare centre
2. Healthcare centre	2. Healthcare centre	6. Churches	1. Markets	5. Municipality
3. Schools	4. Malls	7. Square/ Park	5. Municipality	6. Churches
4. Malls	5. Municipality	5. Municipality	6. Churches	3. Schools

**Table 4:** Desired laboratory parameters* from point-of-care testing device

Regional decision-makers	Urban setting (PHCW)	Rural setting (PHCW)
1. Glucose	1. Glucose	1. Glucose
2. Triglycerides	4. HbA1c	4. HbA1c
3. Urea/Creatinine	5. Cholesterol	5. Cholesterol
4. HbA1c	2. Triglycerides	3. Urea/Creatinine
5. Cholesterol	3. Urea/Creatinine	2. Triglycerides
6. GOT/GPT transaminases	6. GOT/GPT transaminases	8. Electrolytes
7. Thyroid profile	8. Electrolytes	6. GOT/GPT transaminases
8. Electrolytes	7. Thyroid profile	7. Thyroid profile

*Desired parameters were not asked to community members. PHCW: Primary healthcare workers; HbA1c: glycated haemoglobin; GOT/GPT: oxaloacetic transaminase/glutamic pyruvic transaminase

### Proposed implementation strategies

The proposed strategy for implementation of multiparameter POCT devices includes:

(i) *Training*: To train health personnel in the diagnosis and identification of risk factors for NCDs and in the use and management of the POCT device. Community health workers will also be trained in identifying risk factors for NCDs.
(ii) *Location of POCT device*: The POCT device will be placed in the laboratory area where routine laboratory tests are performed.
(iii) *Laboratory parameters*: The selected device should be capable to perform tests for glucose, glycated haemoglobin, cholesterol, and creatinine.
(iv) *Advertisement*: Advertising must focus on raising awareness about POCT device and the acceptability of the devices by the community. Advertising must be placed at the healthcare centre and the municipality using billboards and flyers, also messages through megaphones could be delivered.

## Discussion

### Main findings

The study included three groups of stakeholders involved in the process of care, diagnosis, and treatment, including: the regional decision-makers, healthcare providers, and end-users (community members). The current study found that community members typically require at least three days to receive a diagnosis for an NCD and receive treatment. In an ideal scenario, they would prefer to receive their laboratory test results and receive care within a single day. The PHCW highlighted the need for laboratory test parameters that are not commonly available due to a lack of supplies (e.g. glucose) and those parameters that are not available at the primary health care level (e.g. glycated haemoglobin). Adequate advertising in the community and training for PHCW in the use of the device are necessary for future implementation. The needs mentioned by the different stakeholders could be a response to a weak primary healthcare level or a low level of satisfaction between the patients [22, 23].

### Co-creation for the development of interventions

Methods such as co-creation, which aim for developing interventions with and for different stakeholders, are relevant in the field of implementation science. The value of implementation science involves not only knowing what to do; it also focuses on how to effectively bring theory to reality [24, 25]. For this reason, the process followed and described in the current manuscript is relevant as it provides a practical example of designing an intervention for the implementation of POCT devices. Similar examples have been presented in previous studies. For instance, a co-creation study involving members of the POCT device manufacturing industry found that the use of POCT devices in pharmacies could reduce medical consultation time and reduce costs [26]. However, similar to our study, concerns were raised regarding the use of POCT devices outside the healthcare facility and/or by other stakeholders (community health workers or pharmacist), as well as legal and logistical barriers. A systematic review found that the use of POCT devices in pharmacies could reduce total cholesterol during follow-up; however, it did not show differences in the use of POCT devices for glycated haemoglobin [27]. On the other hand, a study showed that physicians interviewed were not interested in using POCT device themselves, because if testing took considerable amount of time it was better to refer patients to another area [28]. Nevertheless, these studies had the limitation of not including end users’ opinions; and using only personal interviews.

The process of co-creation includes establishing the objective identifying and end-users, the selection of the participants (co-creators), co-creation sessions’ activities, the evaluation of results, and planning the next steps of implementation [18]. We followed these steps because involving different stakeholders and considering the context in which it will be developed can promote the sustainability and scalability of the implementation strategy.

### POCT device in LMIC and PHC settings

Evidence available highlights the importance of ensuring that POCT devices are of high-quality, cost-effective, accessible, and require minimal maintenance and training to be used by PHCW [29, 30]. Our findings showed that participants were a concerned about the training required to use the device and the maintenance needed. The implementation of multiparameter POCT devices at the primary healthcare level can be challenging. The use of POCT devices at the PHC has shown cost reduction by avoiding unnecessary emergency consultations [31]. The implementation of POCT devices in a tertiary hospital included three stages of training for the staff: initial formal POCT device training, recertification, and competency assessment [29]. An initial certification and retraining were also described as necessary in our findings. In many LMICs, the infrastructure and available resources for POCT devices are limited, including issues such as power outages and a lack of equipment (e.g. refrigerators for reagents) [32]. Also, the environmental conditions are less predictable, which can affect the performance and accuracy of POCT device. Two POCT devices for glycated haemoglobin were assessed in the jungle of Peru, and it was found that barometric pressure can modify the results, but not humidity or temperature [13]. However, even though POCT devices perform acceptably under realistic clinical conditions, further evaluation of their performance in the clinical setting is necessary for future implementation [9]. All these considerations need to be contemplated in the development of strategies for using multiparameter POCT devices and ensuring the best implementation in the health system.

### Relevance for public health

The participation of various stakeholders, including patients, health personnel, and regional decision-makers offers a comprehensive approach to understanding the real needs of the population and considering diverse perspectives and potential solutions. The co-creation approach can be applied to other types of conditions (e.g. communicable diseases) or devices to implement context-specific interventions adapted to different groups (e.g. rural vs. urban, different age groups, etc.) and ultimately improve health outcomes [17].

### Recommendations after the experience

Working with groups in the community can lead to delays, so flexibility should be allowed. In addition, keep in mind that there may be changes of authorities between the different workshops, so the research team must keep in touch with the key actors in the sites. We believe it is important to maintain a local collaborator at the study site to facilitate communication with the participating centres.

Another recommendation is to map whether there are key members in the community who can facilitate the recruitment of participants. Community health workers had a great influence on the participation of community members in both settings, which was unexpected by the research team but critical for the development of the workshops. The importance of voluntaries’ participation has been seen in previous studies, where volunteers staff such as “*Promotoras*” or “*Amigas de Liz*” participated in door-to-door recruitment or surveying community members or promoting health activities; resulting in an increased response rate or satisfaction in participants [33, 34].

In addition, other factors that can affect the participation of stakeholders and the implementation process, such as social and cultural issues, the political context, or the preparation of the community should be taken into account [33, 35].

### Limitations and strengths

This study has some limitations that should be mentioned: First, the workshops were conducted with a group of participants, not all of whom were familiar with NCDs and the challenges faced by patients with these conditions. Second, the participation in the groups was not homogeneous, some participants were more talkative than others. However, having small groups helped the facilitators promote the sharing of opinions among most participants, facilitating greater interaction. Lastly, there was no consensus meeting with all the different key stakeholders (regional decision-makers, PHCW and community members). As a strength of our study, we found that the context of limited access to laboratory tests in the studied settings contributed to the high expectations about the potential implementation of POCT devices. This supports a strong acceptance of POCT devices for future implementation.

## Conclusion

The current study described the findings of a co-creation process that began with a scoping review identifying barriers and facilitators for implementing POCT devices and concluded with the development of an intervention ready to be implemented. The co-creation process was useful to develop strategies for the implementation of multiparameter POCT devices for NCDs, involving the participation of different groups of stakeholders guided by moderators in both, rural and urban, settings in Peru.

### Electronic supplementary material

Below is the link to the electronic supplementary material.

Supplementary Material 1

## Data Availability

Data and reports used and analysed during the current study are available from the corresponding author upon reasonable request.

## Abbreviations

NCDs: Non-communicable diseases
LMICs: Low-and middle-income countries
POCT: Point-of-care testing
PHCs: Primary healthcare centres
PHCW: Primary healthcare workers
