# Supplementary material for: Co-creation process of an intervention to implement a multiparameter point-of-care testing device in a primary healthcare setting for non-communicable diseases in Peru
Source: BMC Health Serv Res. 2024 Mar 29;24:401. doi: 10.1186/s12913-024-10809-3 (PMC10981306; doi:10.1186/s12913-024-10809-3)
Supplement: Supplementary file 1 — Supplementary Material 1 [file 12913_2024_10809_MOESM1_ESM.docx]

**Supplementary material**

**Supplementary table 1.** Characteristics of participants in the co-creation process.

| **Variable** | **n (%)** |
| --- | --- |
| **Sex** |  |
| Female | 63 (80.8%) |
| Male | 15 (19.2%) |
| **Age, mean ± SD** | 57.1 ± 15.4 |
| **Group** |  |
| Decision maker | 8 (10.3%) |
| Community member | 59 (75.6%) |
| Health worker | 11 (14.1%) |
| **Any chronic diseases** |  |
| No | 10 (19.6%) |
| Yes | 41 (80.4%) |
| **Diabetes** |  |
| No | 29 (56.9%) |
| Yes | 22 (43.1%) |
| **Hypertension** |  |
| No | 28 (54.9%) |
| Yes | 23 (45.1%) |
| **Dyslipidemia** |  |
| No | 40 (78.4%) |
| Yes | 11 (21.6%) |
| **Chronic kidney disease** |  |
| No | 48 (94.1%) |
| Yes | 3 (5.9%) |
| **Coronary heart disease** |  |
| No | 49 (98.0%) |
| Yes | 1 (2.0%) |
| **Other chronic diseases** |  |
| No | 47 (94.0%) |
| Yes | 3 (6.0%) |
